# Psychosocial Impact of Quarantines: A Systematic Review with Meta-Analysis

**DOI:** 10.3390/healthcare12232409

**Published:** 2024-11-30

**Authors:** Catarina Fabiola González González, Marcelo Navarro, Fabiola María del Carmen Helbig Soto, Braulio Henrique Magnani Branco, Daniela Avello, Florencia Carmine, Nicolás Márquez Álvarez, Cristian Sandoval, Síbila Floriano Landim, Marcelo Leiva-Bianch

**Affiliations:** 1 Facultad de Psicología, Universidad de Talca, Talca 3465548, Chile; cgonzalezg@utalca.cl (C.F.G.G.); navarro.marcelo.f@gmail.com (M.N.); marcleiva@utalca.cl (M.L.-B.); 2 Escuela de Terapia Ocupacional, Facultad de Psicología, Universidad de Talca, Talca 3465548, Chile; fabiola.helbig@utalca.cl; 3 Graduate Program in Health Promotion, Cesumar University (UniCesumar), Maringá 87050-900, Brazil; braulio.branco@unicesumar.edu.br; 4 Departamento de Terapia Ocupacional, Escuela de Ciencias de la Salud, Facultad de Medicina, Pontificia Universidad Católica de Chile, Santiago 7820436, Chile; daniela.avello@uc.cl; 5 Centro de Desarrollo de Tecnologías de Inclusión (CEDETI UC), Pontificia Universidad Católica de Chile, Santiago 7820436, Chile; 6 Carrera de Medicina, Facultad de Medicina, Universidad de La Frontera, Temuco 4811230, Chile; f.carmine02@ufromail.cl; 7 Escuela de Ingeniería Comercial, Facultad de Economía y Negocios, Universidad Santo Tomás, Talca 3460000, Chile; nmarquez4@santotomas.cl; 8 Escuela de Tecnología Médica, Facultad de Salud, Universidad Santo Tomás, Osorno 5310431, Chile; 9 Departamento de Medicina Interna, Facultad de Medicina, Universidad de La Frontera, Temuco 4811230, Chile; 10 Núcleo Científico y Tecnológico en Biorecursos (BIOREN), Universidad de La Frontera, Temuco 4811230, Chile

**Keywords:** isolation, meta-analysis, psychosocial impact, quarantine, social distancing

## Abstract

**Background**: Quarantine is one of the most effective strategies to control outbreaks of communicable diseases. Individuals under isolation or quarantine experience both physical and mental effects. Therefore, given its widespread implementation around the world, it is pertinent to analyze this effect on physical and mental health. The psychosocial impact model, which divides four dimensions into two main points, exposure or protection and positive or negative responses, was used to analyze the psychosocial impact of quarantine. **Objectives**: The present study aimed to investigate the psychosocial impact of people exposed to or protected from quarantine. **Methods**: We conducted a search for primary articles in the Web of Science and Scopus databases, and after applying the inclusion and exclusion criteria, we meta-analyzed five of them. **Results**: Quarantined individuals were more likely to experience anxiety (*K* = 4; *OR* = 2.62) and depressive symptoms (*K* = 6; *OR* = 1.61) compared to those who did not undergo quarantine. Researchers discuss a twofold increase in the probability of anxiety or depression among those in quarantine. As a result, advancing interventions that reduce this impact is critical for both health and the global economy. **Conclusions**: In terms of economic variables, the non-moderation of GDP per capita and the moderation of the GINI index stand out, demonstrating that countries must move toward policies that promote the reconstruction of more resilient and inclusive societies.

## 1. Introduction

In December 2019, Wuhan, China’s Hubei province, recorded a number of severe pneumonia cases for no apparent reason [1]. The study of these cases revealed that the cause of this pneumonia was a new coronavirus strain that shared similarities with the four human coronaviruses that caused the common cold, Middle East respiratory disease (MERS), and severe acute respiratory syndrome (SARS). The WHO proclaimed the SARS-CoV-2 outbreak to be a pandemic on 30 January 2020 [2].

A coronavirus causes COVID-19, an infectious disease that spreads through salivary droplets or nasal discharge [2]. It has an infectious average (R^0^) that ranges between 2.24 and 3.58 [3], which means that it is very contagious. Given the accelerated spread of the virus, various localities, cities, and countries implemented measures and public policies that sought to restrict social contact. The most common measure is population quarantine [4].

China declared quarantine in several main cities, such as Wuhan [5]. In Europe, Italy was the country with the most infections at the beginning of the coronavirus, implementing restrictions on attendance in public places and promoting physical distancing and community quarantine [6,7]. Brazil registered approximately nine million infections, becoming the Latin American country with the most cases by the end of 2020 [8].

COVID-19 generated significant consequences for global health and the economy. The WHO identified COVID-19 as a priority disease for research, highlighting its potential for epidemics and the lack of adequate countermeasures [9]. Likewise, it generated a severe stagnation in the economic activity of various countries, given the suspension or slowing down of product supply chains and the decrease in production in the main economies, among others. The global Gross Domestic Product (GDP) experienced a roughly 3% decrease [10]. Finally, forecasts for 2020 projected a contraction in Latin America and the Caribbean at an average of −5.3% amidst a continuous 7-year decline in low economic growth [11].

Restriction measures, such as quarantine, provide benefits in terms of infection control [12]. However, the literature addresses the existence of mental health consequences derived from prolonged periods of isolation [13,14]. Individuals who experience quarantine are at high risk of developing negative psychological effects, including post-traumatic stress symptoms, confusion, and anger [15]. They also identified a variety of other psychological responses to quarantine, such as confusion [16,17], fear [16,18,19], and insomnia [18,19]. However, not all factors are negative; during quarantine, such aspects as valuing life, experiencing new positive experiences, and discovering new abilities or hobbies, among others, may arise [20].

Research indicates that young people have faced heightened mental health challenges due to the disruption of social and educational routines, leading to feelings of loneliness and uncertainty about the future, also in children and adults [21,22,23,24]. Similarly, studies in Latin America, such as in Bogotá, Colombia, show increased symptoms of depression and anxiety among young adults, exacerbated by economic instability and job loss [25,26]. Healthcare workers have also been deeply affected, experiencing high levels of stress due to intense work demands and the constant risk of infection and their families, compounded by societal stigma [27,28]. Studies from previous outbreaks, including SARS and MERS, show that these impacts can be long-lasting, with extended quarantine leading to social isolation, stigmatization, and post-traumatic stress [29].

Isolation or quarantine has historically impacted people’s health. Given the above, analyzing the impact of this restrictive measure is relevant. For this reason, the WHO recommends the use of systematic reviews and meta-analyses to strengthen health policies and systems [30]. In this context, the current meta-analysis focuses on the psychosocial effects of quarantines. Specifically, the research question guiding this meta-analysis is, “What is the psychosocial impact of quarantine in terms of mental health (such as anxiety, depression, well-being, and resilience), and how does this impact vary according to socioeconomic factors like GDP per capita and the GINI index? We will also analyze whether socioeconomic indicators, such as GDP per capita and the GINI Index, moderate the relationship between exposure to quarantines and the resulting disruptive or healthy responses. This review reveals that no previous study has tackled this topic, making this meta-analysis pertinent. In other words, no study has comprehensively analyzed the potential health or disruption responses that different populations may have to quarantine. Therefore, the objective was to determine the psychosocial impact of quarantine on people and communities. We categorized the studies based on their factors and dependent variables to conduct a meta-analysis [31].

Many articles incorporate the concept of psychosocial impact, but it still lacks a clear definition. For the above, Leiva-Bianchi et al. [31] propose a model to explain how it is associated with a traumatic event. This model proposes two axes: the first corresponds to positive responses (i.e., coping, post-traumatic growth, social support, emotional well-being, and positive emotions) and negative responses (i.e., post-traumatic stress, stress, depression, anxiety, substance abuse, hate responses, disruptive behavior, and general psychopathology). The second axis corresponds to protection versus exposure to the event. This model illustrates four distinct types of psychosocial impact: resilient, where the individual experiences an event and demonstrates healthy or non-disruptive responses; witness, where the individual receives protection from the event and demonstrates healthy responses; sensitive, where the individual receives protection from the event but exhibits disruptive responses; and traumatic, where the individual experiences an event and exhibits negative responses.

## 2. Materials and Methods

### 2.1. Search Strategy

To conduct a meta-analysis on the psychosocial impact of quarantine, we followed the PRISMA guidelines [32,33]. We performed searches in the Scopus and Web of Science databases for articles published up to 31 December 2020. The following search terms were used: “psychosocial impact”; “psychosocial effect”; “psychological impact”; “psychological effect”; “mental health”; “post-traumatic growth”; “well-being”; “resilience”; “quarantine”; “isolation”; “social isolation”; and “social distancing”. To ensure a comprehensive search, we used a total of 32 search combinations and did not impose language or publication year restrictions. Protocol was recorded under the following link: https://doi.org/10.17605/OSF.IO/E9S5P.

#### Study Selection Process and Data Collection

Study selection process and data collection: This study’s selection process involved two independent (SFL, CS) reviewers who screened all identified articles based on predefined inclusion and exclusion criteria. Discrepancies between the reviewers were resolved through discussion, ensuring that only studies meeting the criteria were included. Automation tools were utilized to facilitate the initial screening of titles and abstracts, enhancing efficiency in the selection process.

Data collection was conducted by the same reviewers, who independently extracted relevant data from each study, including sample sizes, means, standard deviations, and measures of association (e.g., odds ratios and correlation coefficients). To confirm accuracy, a subset of studies was cross-checked for data consistency, and any discrepancies were discussed and resolved.

### 2.2. Inclusion and Exclusion Criteria

#### 2.2.1. Studies Were Included If They Met the Following Criteria

To ensure the robustness and relevance of the studies selected for this meta-analysis, specific inclusion and exclusion criteria were applied. These criteria were established based on the objective of analyzing the psychosocial impact of quarantine and ensuring the methodological quality of the included studies.

#### 2.2.2. Inclusion Criteria: Studies Were Included in the Meta-Analysis If They Met All of the Following Criteria

Data type: Only studies that provided quantitative data comparing quarantine-exposed groups with non-exposed groups were considered. Qualitative data, i.e., studies that did not provide measurable results or clear statistical comparisons between groups, were excluded;

Outcome indicators: Studies were required to report at least one relevant psychosocial indicator, such as odds ratios (*OR*), correlation coefficients, means and standard deviations for continuous outcomes, and frequencies of psychological symptoms or events (e.g., anxiety, depression).

These indicators allowed for quantitative analysis and comparison of the effects of quarantine versus no quarantine in the involved populations;

Study Design: Only studies with robust quantitative designs were included, such as cohort studies (prospective or retrospective), randomized controlled trials, case-control studies, or cross-sectional studies that directly compared quarantine-exposed and non-exposed groups;

Population: These studies needed to include an adult population (18 years or older), as the psychosocial impact of quarantine might differ in younger populations or children, who were excluded to avoid developmental bias.

### 2.3. Meta-Analysis Methodology

#### 2.3.1. The Meta-Analysis for This Study Followed These Steps

Data extraction: Relevant data from the studies were extracted, including sample sizes, means, standard deviations, and measures of association (e.g., odds ratios and correlation coefficients). These data were organized to facilitate comparison.

#### 2.3.2. Effect Size Calculation

Standardized mean differences (Cohen’s d): For studies reporting continuous outcomes (e.g., means and standard deviations of symptoms), Cohen’s d was calculated to measure the standardized difference between the exposed and control groups.

Odds ratios: For studies with categorical outcomes (e.g., presence or absence of symptoms), odds ratios were calculated to measure the odds of an outcome occurring in the exposed group relative to the control group.

### 2.4. Statistical Analysis

#### 2.4.1. Model Selection

A fixed-effects model was used when studies were assumed to estimate a common effect size. A random-effects model was applied when there was significant heterogeneity among studies, which was determined using the I^2^ statistic.

#### 2.4.2. Heterogeneity Assessment

The I^2^ statistic was used to assess heterogeneity, with values interpreted as low (≤25%), moderate (26–50%), or high (>50%).

### 2.5. Sensitivity Analysis and Publication Bias

#### 2.5.1. Sensitivity Analysis

Conducted by systematically removing each study one at a time to observe changes in the overall effect size and assess the robustness of the results.

#### 2.5.2. Publication Bias

Evaluated using funnel plots and Egger’s test to detect any potential publication bias that might skew the results.

#### 2.5.3. Meta-Regression

Conducted to evaluate whether external variables, such as GDP per capita and GINI index, moderated the relationship between quarantine exposure and psychosocial outcomes. The meta-regression model was used to analyze the impact of these moderators.

### 2.6. Statistical Techniques Used

#### Effect Size Calculation

Odds ratios: to measure the relative odds of outcomes (such as anxiety and depression) in the exposed versus non-exposed groups.

Standardized mean differences (Cohen’s d): for studies reporting means and standard deviations.

### 2.7. Heterogeneity Testing

#### 2.7.1. I^2^ Statistic

Measures the proportion of total variation across studies due to heterogeneity rather than chance.

#### 2.7.2. Fixed-Effects Model

Used when studies are considered to estimate the same effect size.

#### 2.7.3. Random-Effects Model

Applied when there is significant variability between studies.

#### 2.7.4. Sensitivity Analysis and Publication Bias Detection

Sensitivity analysis: tested by removing individual studies to evaluate their impact on the overall effect size;

Funnel plots and Egger’s test: used to identify and test for publication bias;

Meta-regression: analyzed the moderating effects of GDP per capita and GINI index on the relationship between quarantine exposure and psychosocial outcomes.

## 3. Results

### Selected Articles

**Figure 1:**
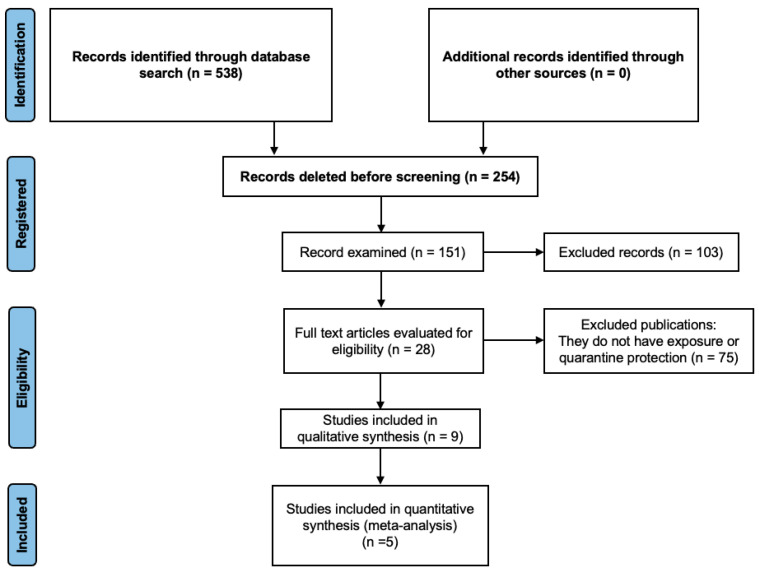
A scientific literature selection process.

A total of 538 articles were found. After applying the inclusion criteria, this study was reduced to five studies. The information from each article was extracted and sorted according to the surname of the first author, country of publication, extraction database, publication journal, year of publication, sample, identified risk factors, and meta-analyzed statistics. Table 1 identifies these articles, while Figure 1 illustrates the general selection process for the search of the scientific literature.

A total of 538 articles were initially identified through our search strategy. After screening titles and abstracts for relevance, we applied the predefined inclusion and exclusion criteria, which led to the exclusion of studies that did not meet the requirements. Specifically, studies were excluded based on factors such as the lack of quantitative data, non-relevant outcomes, or failure to compare quarantine-exposed populations with non-exposed populations. Following this, full-text articles were assessed for eligibility. Ultimately, after the full-text review and applying the final set of inclusion criteria, five studies were selected for inclusion in the meta-analysis.

The extracted data from these five studies were organized and categorized by the following variables: the surname of the first author, the country of publication, the database used for extraction, the publication journal, the year of publication, the sample characteristics, identified risk factors, and the statistics used for meta-analysis (e.g., effect sizes, odds ratios, etc.).

The prism flow diagram illustrates the process of study selection, from the initial search results to the final inclusion of studies. Each stage of the screening process, including the number of studies excluded at each step, is represented in the flowchart to enhance transparency in the selection process.

All statistics found in the studies were transformed into *OR* values and the variance of the logarithm of *OR* using the rECSMA software [39]. To analyze the moderating effect of the GDP per capita variable and the GINI index, the meta-regression model was used.

Studies on anxiety (*k* = 4) and depression (*k* = 5) were found as indicators of the psychosocial impact of quarantines (Table 2). The effect ranges between −1.24 and 14.23 for anxiety and between −12.07 and 44.12 for depression (Table 3).

**Figure 2:**
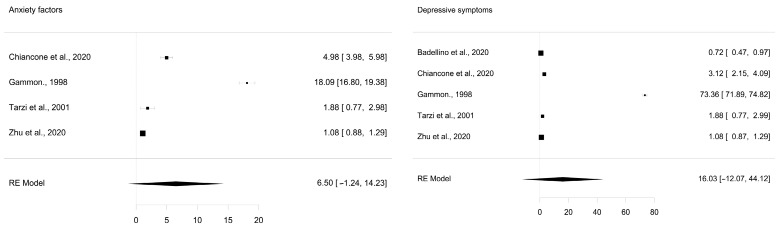
Forest plot of psychosocial impact factors of quarantines [34,35,36,37,38].

Both anxiety and depressive symptoms do not present a statistically significant effect. However, analyzing the forest plot of both factors (Figure 2) reveals deviations in the *ORs* for both factors. To resolve this, a sensitivity analysis was performed to indicate the change in *OR* when removing each of the studies (Table 4). When removing study A2, there is a notable change (*OR* = 6.50; SE = 3.95; *p* = 0.100; CI = [−1.237–14.229]) to A2 (*OR* = 2.62; SE = 1.19; *p* < 0.05; CI = [0.028–4.951]). In other words, quarantined people are twice as likely to have anxiety. On the other hand, when removing the D3 study, there is a notable change from (*OR* = 16.0; SE = 14.3; *p* = 0.264; CI = [−12.068–44.120]) to D3 (*OR* = 1.6; SE = 0.522; *p* < 0.05; CI = [0.587–2.635]). This way, depressive symptoms would be twice as likely among those in quarantine as among those who are not. Note that the withdrawn study on anxiety and depression aligns with Gammon et al.’s [36] findings. This study exposed the sample not only to quarantine but also to various diseases such as methicillin-resistant *Staphylococcus aureus*, *Clostridium difficile*, and *Salmonella typhimurium*. As a result, these diseases may alter levels associated with depressive symptoms and anxiety.

Heterogeneity tests and the Rosenthal test for anxiety and depression both showed high rates of intra-study heterogeneity. Rosenthal’s NS was high for all factors, indicating that the potential number of studies with a null effect should be extremely higher than the articles presented in this study. Finally, the meta-regression analysis indicates that GDP would not be a moderating variable in the higher levels of depression in populations exposed to quarantines. However, the GINI index acts as a significant moderator for depressive symptoms (Table 5). The results indicate a significant and positive moderating effect of the inequality indicator on the relationship between quarantine exposure and depression. In other words, in those countries with greater inequality, people exposed to quarantine would have more depressive symptoms than in those countries with less inequality. The effect of inequality in moderation is high (around 56%).

## 4. Discussion

This study’s main finding is that the probability of having anxiety or depression increases twice as much among those in quarantine. A closer look at particular components related to quarantine uncovers critical aspects that result in the heightened symptoms of anxiety and depression. Social isolation is compounded by the absence of physical and emotional touch from friends and family, leading to feelings of loneliness and alienation [15]. Directly correlated to emotional stability, fears over losing income or jobs are a principal cause of increased anxiety and depression [39]. Mobility and loss of freedom restrictions also restrict the opportunities envisioned to cope, for example, exercise or outdoor activities that are recognized as stress and anxiety protectors [40]. Furthermore, 24/7 news coverage of infections and deaths from the pandemic contributes to information overload, leading to an increased hyper-arousal state of anxiety [41]. Such factors underscore the importance of developing interventions not only for mitigating infection but also for addressing many of the psychosocial effects of quarantine.

Immediate measures such as integrating mental health services within national emergency response frameworks can provide essential support during quarantines [42]. Additionally, the implementation of telehealth services and online cognitive–behavioral therapies ensures the continuity of mental health care despite physical distancing measures [43]. To mitigate feelings of isolation, policies promoting virtual social interactions and community engagement activities are necessary [44]. These can provide emotional support and reduce the psychological burden associated with social isolation [15]. Regular mental health screenings can facilitate early detection and management of anxiety and depression, ensuring timely intervention for those at risk [45]. Economic support for individuals most affected by quarantines can also alleviate the financial stresses that contribute to mental health issues [46]. Furthermore, enhancing public awareness about the psychological impacts of quarantine through education and public campaigns can help reduce stigma and encourage individuals to seek mental health care proactively [47].

Previous studies confirm that anxiety [18,19,48,49] and depressive symptoms [50,51] are among the most commonly reported and studied psychological impacts during quarantines. However, our meta-analysis indicates that, despite the focus on these symptoms, there has been a lack of large-scale, comprehensive investigations specifically comparing the psychosocial effects of quarantine across different populations. The study of Henssler et al. [52] validates the present study’s findings by concluding that people experiencing quarantine have a higher risk of developing depression, anxiety, stress-related disorders, and anger.

From the information processing perspective, anxiety is an emotional outcome of complex biopsychosocial responses with significant evolutionary, biological, affective, and cognitive components [53]. The latest research suggests that screening for anxiety and depressive symptoms in acute quarantine contexts may be a temporary, functional response to an extreme situational stressor rather than an indicator of chronic psychiatric disorders [45]. Intense, transient stressors, such as social isolation and concerns about health [54], frequently manifest as elevated anxiety, depression, and disrupted sleep. Although these responses may be distressing, they may also dissipate as the effects of normal life resume [55]. It is vital to understand this distinction, as it emphasizes that acute symptoms should not be equated with a diagnosis of an anxiety or depressive disorder. Furthermore, it facilitates a nuanced interpretation of the mental health effects following quarantine.

Furthermore, depression is one of the most prevalent and severe psychiatric disorders worldwide [56]. In addition, people are less likely to have adequate coping skills and, therefore, become depressed when faced with a problem [44]. Therefore, it is necessary to support interventions that focus on developing these skills, not only as a way to treat depression but also to prevent its recurrence [42]. Implementing interventions such as Beck’s short-term cognitive therapy, which focuses on understanding idiosyncratic dysfunctional beliefs, specific vulnerabilities based on distorted latent schemes, and specific stressful events, is necessary to achieve this [43].

To provide a deeper understanding of our findings, we compared them with recent cross-sectional and longitudinal studies examining anxiety and depressive symptoms in populations not subjected to quarantine during the pandemic. For instance, Ettman et al. [47] reported a significant increase in depressive symptoms across the general population, attributed to widespread factors such as economic uncertainty, disruptions in daily life, and health-related concerns, even among those not quarantined. Similarly, a longitudinal study by Pierce et al. [57] observed a sharp rise in anxiety and depression levels within the general UK population in the early months of the pandemic, irrespective of isolation status, suggesting that broader pandemic-related stressors played a substantial role [57]. By contrasting these findings with our own, we aim to clarify the unique impact of quarantine itself, distinguish it from the general psychosocial effects linked to the pandemic, and identify specific factors that may elevate mental health risks under quarantine conditions.

Depression and anxiety disorders are common mental health problems that affect work capacity and productivity. More than 300 million people worldwide suffer from depression, and more than 260 million have anxiety disorders [58]. To achieve this goal, the WHO states that every dollar invested in expanding treatment for depression and anxiety yields four dollars in improved health and work capacity [59]. Therefore, investing in these treatments is of utmost importance for global policies.

There is incipient research on the moderating variables reviewed in this study: the GINI index and GDP per capita. In Peru, researchers analyzed a model that established a relationship between an epidemic, public policy, and the level of economic activity, concluding that, in the medium term, a partial or rigid quarantine might benefit the economy more than its absence [60]. Lockdowns themselves may not present a clear trade-off between GDP and public health, either. A hasty lifting of quarantine momentarily raises GDP. However, infections increase over time. Likewise, low-skilled workers and the self-employed are always the ones who lose the most from both the pandemic and containment policies [46]. Similarly, economic growth creates large social gaps and increases long-term environmental costs [61]. Finally, it is important to note that half of the articles analyzed in this study present a sample with an average age greater than 80 years. Therefore, this could indicate a lack of close linkage to any economic activity, leading to no clear moderation of GDP in this meta-analysis.

The GINI index had a significant and important impact. Economic inequality in quarantine settings accounts for approximately 56% of the increase in depression. Therefore, in countries where inequality is greater, this relationship becomes more relevant. From the perspective of mental health, the important thing would be to move toward equality and not so much toward economic growth. State measures that consider an improvement in the conditions of equality would have better effects on people exposed to quarantines. Since 2015, the United Nations (UN) has been working on the 2030 Agenda for Sustainable Development, organizing interregional meetings to discuss equality and its significant challenges [62]. The COVID-19 pandemic caused the worst economic and social crisis in Latin America and the Caribbean in decades, with detrimental effects on growth, poverty, employment, and the reduction in inequality. Therefore, policies to rebuild more resilient and inclusive societies in the post-pandemic phase are of extreme importance [11].

Research on the impact of quarantines, both positive and negative, is scarce. According to Leiva-Bianchi et al. [31], the articles forming the basis of this study primarily focus on the sensitive and traumatic quadrants. Because there is little research, they do not discuss resilience or witness quadrants where quarantine generates improvements in people. Therefore, future studies need to explore the positive aspects of quarantine in the medium term.

### 4.1. Psychosocial Interventions and Policy Implications to Mitigate Quarantine-Related Impacts

The findings of this meta-analysis emphasize the significant psychological toll of quarantine on individuals, particularly in terms of increased anxiety and depression. While cognitive therapy and broader policy considerations are acknowledged, it is crucial to further explore concrete psychosocial interventions and more specific policy actions that can counterbalance these effects and support mental health during times of isolation.

#### 4.1.1. Psychosocial Interventions to Address Anxiety and Depression

Given the heightened risk of anxiety and depression associated with quarantine, several interventions have shown promise in mitigating these effects. These include therapeutic models, community-based programs, and mental health support initiatives that can be deployed both at the individual and societal levels.

#### 4.1.2. Cognitive Behavioral Therapy (CBT)

As a well-established approach for treating anxiety and depression, CBT focuses on helping individuals identify and change negative thought patterns and maladaptive behaviors. In the context of quarantine, digital or online CBT interventions are highly effective, as they allow people to access therapy remotely, maintaining continuity of care even during periods of isolation [43]. The use of digital platforms to administer CBT could be a particularly useful strategy for broadening access to mental health services during emergencies like the COVID-19 pandemic.

#### 4.1.3. Mindfulness-Based Stress Reduction (MBSR)

Mindfulness interventions, such as MBSR, have been found to reduce stress and improve emotional regulation, which is especially important during times of crisis [53]. These practices, when implemented remotely or in online formats, can provide a valuable resource for individuals struggling with the psychological impacts of quarantine, helping them cope with stress, anxiety, and depressive symptoms [56].

#### 4.1.4. Community Support Networks

Strengthening social support networks is critical for reducing feelings of isolation, which have been shown to exacerbate mental health issues during quarantine [44]. Online support groups, peer counseling, and virtual community-building initiatives can provide much-needed social interaction and emotional support, helping individuals feel connected even when physically distanced.

#### 4.1.5. Psychoeducation and Mental Health Literacy

Providing education on mental health issues, particularly related to anxiety and depression, can empower individuals to recognize early signs of distress and seek appropriate help [42]. Psychoeducation programs can be delivered through online platforms, mobile apps, or public service campaigns, ensuring widespread access and reducing the stigma associated with seeking mental health care.

#### 4.1.6. Policy Implications for Mental Health Support During Quarantines

Governments and public health organizations must recognize the psychosocial impact of quarantines and integrate mental health support into their public health response plans. Specific policy measures should be implemented to protect and promote the psychological well-being of individuals during quarantine:

#### 4.1.7. Incorporating Mental Health Services in Emergency Responses

Mental health services should be integrated into national and local emergency response plans to ensure that individuals have access to psychological support during periods of quarantine. Telehealth services, hotlines, and online counseling should be made widely available, offering immediate and easily accessible support for those in need [59].

#### 4.1.8. Regular Psychological Monitoring and Early Intervention

Governments should implement systems for regular mental health screening and monitoring during quarantines, particularly for vulnerable populations. Digital mental health surveys or phone consultations can help identify individuals at risk of developing anxiety, depression, or other mental health issues early, enabling timely intervention [58].

#### 4.1.9. Community-Based Initiatives to Combat Isolation

Policymakers should encourage the development of community-based initiatives that combat social isolation during quarantine. This could involve facilitating virtual social activities, supporting the creation of online social networks, or funding community mental health programs that promote well-being through collective action [58].

#### 4.1.10. Workplace Mental Health Support

During times of quarantine, individuals who continue to work remotely may experience increased stress and reduced mental well-being. Policies to support mental health at the workplace, such as providing access to virtual counseling services, promoting flexible working hours, and encouraging stress management practices, can help reduce the psychological burden on employees [59].

### 4.2. Future Directions

While cognitive therapies like CBT and mindfulness programs are essential in addressing the mental health impact of quarantine, the implementation of comprehensive and accessible psychosocial interventions, alongside well-crafted public health policies, is necessary for mitigating long-term psychological effects. Future research should explore the effectiveness of these interventions across different settings and populations, as well as evaluate the role of public policy in providing sustainable mental health support during health crises.

### 4.3. Scope and Limitations

Although the above findings improve our understanding of the impact of quarantine, this meta-analysis has limitations. Firstly, the meta-analysis only encompassed articles from two databases, excluding studies that could potentially expand our search and enhance the clarity of our results. Furthermore, both categories consisted of fewer than five articles, which could limit the generalizability of the findings. However, several researchers reviewed this study’s construction, establishing well-designed and defined exclusion and inclusion criteria.

Secondly, the inclusion and exclusion criteria presented in this study omitted several studies that could have produced more information. To remedy this, when performing a simple search in the databases analyzed in this study, 2020 and the word “quarantine” were considered the limit. We found a total of 19 articles for the anxiety factor and 14 for the depression variable, and after eliminating duplicates, we identified 15 articles suitable for further meta-analysis (Table 6).

## 5. Conclusions

It is crucial to emphasize that this research serves as a platform for other researchers to explore and shed light on the psychosocial effects of quarantine, particularly considering that quarantines will likely become more common practices in the future. Finally, it is important to note that anxiety and depression are not the “factors” of psychosocial impact but rather the two variables that our review uses as responses.

## Figures and Tables

**Table 1:** Articles included in the meta-analysis.

ID	Reference	Country	GINI Index	GDP per Capita	Method	Instrument	N	Women	SD
1	[34]	ARG	42.9	9912.3	Cross-sectional study through a digital questionnaire	GAD-7, PHQ-9, PSS, and PSQI.	1985	75.9%	36.83 ± 14.41
2	[35]	IT	35.9	2099.6	Retrospective data review	GAD-7, PHQ-9, NRS, and VAS.	56	N.I.	N.I.
3	[36]	UK	37	28,214.3	Quasi-experimental study	HADS, Self-esteem Scale, and Health-Illness.	40	40%	56 years
4	[37]	UK	37.3	27,744.5	Cross-sectional matched control study	BI, GDS, and PDMS.	42	73.8%	80
5	[38]	CN	38.5	10,261.7	Cross-sectional study through a digital questionnaire	SRQ-20, GAD-7, and PHQ-9.	2279	59.7%	N.I.

BI: Barthel Index; GAD-7: Generalized anxiety disorder-7 scale; GDS: Geriatric depression scale short form; N.I.: No information; NRS: Numerical rating scale; PDMS: Profile of mood states; PHQ-9: Patient health questionnaire; PSQI: Pittsburgh sleep quality Index; PSS: Perceived stress scale; VAS: Visual analogic scale. GDP per capita and GINI index values extracted from the Central Bank’s website. For values not found for the Gini Index, the closest value was used.

**Table 2:** Summary of variables that assess the psychosocial impact of quarantines.

Anxiety (A; *k* = 4)					
ID	Author	n	Variable	*OR*	CI (95%)
A1	[35]	56	Anxiety	4.98	[3.98–5.98]
A2	[36]	40	Anxiety	18.09	[16.8–19.38]
A3	[37]	42	Anxiety	1.88	[0.77–2.98]
A4	[38]	2279	Anxiety	1.08	[0.88–1.29]
D1	[34]	1985	Depression	0.72103	[0.47–0.97]
D2	[35]	56	Depression	3.11878	[2.15–4.09]
D3	[36]	40	Depression	73.3564	[71.89–74.82]
D4	[37]	42	Depression	1.87878	[0.77–2.99]
D5	[38]	2279	Depression	1.07977	[0.87–1.29]

*k*: number of articles that make up each factor; n: sample size of each study; *OR*: Odds ratio; CI: Confidence intervals.

**Table 3:** Psychosocial impact factors of quarantine without sensitivity analysis.

	REM						Heterogeneity			Rosenthal Test	
Factor	*OR*	SE	Z	*p*	CI (95%)		I^2^	Q	*p*	NS	*p*
Anxiety	6.50	3.95	1.65	0.10	−1.237	14.229	99.7%	694.642	<0.001	950	<0.001
Depression	16.0	14.3	1.12	0.26	−12.068	44.120	100%	9272.488	<0.001	5625	<0.001

*OR*: Odds ratio; SE: Standard error; CI: Confidence intervals; I^2^: Heterogeneity index; Q: Cochran test; *p*: Level of significance; NS: Safety number.

**Table 4:** Sensitivity analysis of anxiety factors and depressive symptoms.

	REM						Heterogeneity			Rosenthal Test	
ID	*OR*	SE	Z	*p*	CI (95%)		I^2^	Q	*p*	NS	*p*
A1	7.01	5.54	1.27	0.206	−3.845	17.858	99.79%	650.325	<0.001	619	<0.001
A2	2.62	1.19	2.20	0.028	0.287	4.951	96.41%	57.486	<0.001	199	<0.001
A3	8.04	5.15	1.56	0.118	−2.038	18.112	99.78%	694.494	<0.001	831	<0.001
A4	8.31	4.97	1.67	0.094	−1.423	18.043	99.56%	381.442	<0.001	606	<0.001
D1	19.9	17.8	1.11	0.266	−15.102	54.811	99.99%	9153.165	<0.001	5128	<0.001
D2	19.3	18.0	1.07	0.286	−16.086	54.595	100%	9265.594	<0.001	5068	<0.001
D3	1.61	0.52	3.08	0.002	0.587	2.635	96.33%	25.823	<0.001	234	<0.001
D4	19.6	17.9	1.09	0.275	−15.585	54.713	100%	9272.481	<0.001	5327	<0.001
D5	19.8	17.9	1.11	0.269	−15.253	54.783	99.99%	9158.115	<0.001	4737	<0.001

*OR*: Odds ratio; SE: Standard error; CI: Confidence intervals; I^2^: Heterogeneity index; Q: Cochran test; *p*: Level of significance; NS: Safety number; A1: Values when withdrawing an article A1; A2: Values when withdrawing article A2; A3: Values when withdrawing article A3; A4: Values when withdrawing article A4; D1: Values when withdrawing article D1; D2: Values when withdrawing article D2; D3: Values when withdrawing article D3; D4: Values when withdrawing article D4; D5: Values when withdrawing article D5.

**Table 5:** Mixed-Effects Model (MEM) of the depressive symptomatology factor.

Indicator	*OR*	SE	Z	*p*	Mod	SE_Mod_	R^2^	*p*_Mod_
GDP per cápita	1.91	1.04	1.830	0.067	−2.23 × 10^−5^	6.90 × 10^−5^	0%	0.747
GINI	12.377	5.350	−2.03	0.042	−0.277	0.136	56.3%	0.002

*OR*: Odds ratio; SE: Standard error; Mod: Moderator variable.

**Table 6:** Database articles associated with anxiety, depression, and quarantine.

References	Title	Journal	DOI
[63]	Lockdown, quarantine measures, and social distancing: associations with depression, anxiety, and distress at the beginning of the COVID-19 pandemic among adults from Germany.	Psychiatry Res.	10.1016/j.psychres.2020.113462
[64]	The enemy who sealed the world: effects of quarantine due to COVID-19 on sleep quality, anxiety, and psychological distress in the Italian population.	Sleep Med.	10.1016/j.sleep.2020.05.011
[65]	The Relationship Between Media Involvement and Death Anxiety of Self-Quarantined People in the COVID-19 Outbreak in China: The Mediating Roles of Empathy and Sympathy.	Omega-J. Death Dying	10.1177/0030222820960283
[66]	E-learning: Depression, anxiety, and stress symptomatology among Lebanese university students during COVID-19 quarantine.	Nurs. Forum	10.1111/nuf.12521
[67]	The anxiety and loneliness levels in geriatric population in home quarantine during COVID-19 pandemic in Turkey.	Turkish J. Clinical Psychiatry	10.5505/kpd.2020.04382
[68]	Comparison of Prevalence and Associated Factors of Anxiety and Depression Among People Affected by versus People Unaffected by Quarantine During the COVID-19 Epidemic in Southwestern China.	Med. Sci. Monit.	10.12659/MSM.924609
[69]	Levels and predictors of depression, anxiety, and suicidal risk during COVID-19 pandemic in Argentina: the impacts of quarantine extensions on mental health state.	Psychology Health Med.	10.1080/13548506.2020.1867318
[70]	The impact of the COVID-19 pandemic on mental health: Early quarantine-related anxiety and its correlates among Jordanians.	Eastern Mediterr. Health J.	10.26719/emhj.20.115
[71]	How does the quarantine resulting from COVID-19 impact dental appointments and patient anxiety levels?	Braz. Oral Res.	10.1590/1807-3107BOR-2020.VOL34.0084
[72]	Prevalence, risk factors, and clinical correlates of depression in quarantined population during the COVID-19 outbreak.	J. Affec. Disord.	10.1016/j.jad.2020.06.035
[73]	Effect of COVID-19-induced home quarantine on parental stress and its relationship with anxiety and depression among children in Guilan Province.	Iran. J. Psychiatry Clin. Psychol.	10.32598/ijpcp.26.3402.1
[74]	Depression and coping among COVID-19-infected individuals after 10 days of mandatory in-hospital quarantine, Irbid, Jordan.	Psychol. Res. Behav. Manag.	10.2147/PRBM.S267459
[75]	COVID-19-related depression and anxiety among quarantined respondents.	Psychol. Health	10.1080/08870446.2020.1782410
[76]	Exergames as Coping Strategies for Anxiety Disorders during the COVID-19 Quarantine Period.	Games Health J.	10.1089/g4h.2020.0060
[77]	Lifestyle behavior changes during the COVID-19 pandemic quarantine among 6,881 Brazilian adults with depression and 35,143 without depression.	Cien. Saude Colet.	10.1590/1413-812320202510.2.27862020

Information extracted from Scopus and Web of Science databases.
